# Premiums for Residing in Unfavorable Food Environments: Are People Rational?

**DOI:** 10.3390/ijerph19126956

**Published:** 2022-06-07

**Authors:** Meng Yang, Feng Qiu, Juan Tu

**Affiliations:** 1 School of Public Administration, Zhongnan University of Economics and Law, Wuhan 430073, China; 2 Department of Resource Economics and Environmental Sociology, University of Alberta, Edmonton, AB T6G 2H1, Canada; feng.qiu@ualberta.ca; 3 Economics and Management School, Wuhan University, Wuhan 430072, China; tujuan@whu.edu.cn

**Keywords:** food desert, food swamp, spatial hedonic model, willingness to pay, food environment, GIS

## Abstract

The most extensive research areas in the food environment literature include identifying vulnerable dietary environments and studying how these environments affect eating behaviors and health. So far, research on people’s willingness to pay (WTP) for residing in different types of food environments is limited. Therefore, this study aims to estimate WTP for different types of food environments by using spatial hedonic pricing models. The empirical application applies to the Canadian city of Edmonton. The results show that people are willing to pay a premium to live in neighborhoods with poor access to supermarkets and grocery stores (food-desert type) and neighborhoods with excessive access to fast-food restaurants and convenience stores (food-swamp type). Why do rational people prefer to live in disadvantaged food environments? The seemingly counter-intuitive result has its rationality. The premium paid to live in food-desert type environment may reflect people’s dislike of noise, traffic jams, and potential safety issues brought by supermarkets and grocery stores. The WTP for living in food-swamp type environment may reflect people’s preference for convenience and time-saving brought by fast-food consumption in modern urban society. Additionally, the inability of low-income families to afford healthy food may be a deeper reason for choosing to live in neighborhoods with excess access to fast food. To improve the eating environment and encourage healthy lifestyles, the government can encourage healthier fast-food restaurants, provide grocery shopping vouchers, and promote community garden projects.

## 1. Introduction

A person’s diet is closely related to their health [1,2,3]. Given the importance of the effects of diet on health and the relationship between nutrition and various diseases, there is a great deal of research dedicated to examining the relationships between food environments and residents’ dietary behaviors. For example, studies have observed that residents with better access to healthy food sources tend to have more nutritional food intake and lower obesity rates [4,5,6]. In contrast, residents with abundant access to unhealthy food sources tend to consume more fast food and have higher obesity rates [4,7,8].

Researchers are also devoted to identifying unfavorable food environments to help target vulnerable individuals and communities needing emergency assistance. The food environment assessment literature identifies “food deserts” and “food swamps” as the two most widely studied areas. Food deserts often refer to places (e.g., communities or neighborhoods) where healthy food is not easily accessible [9,10,11]. In contrast, Food swamps refer to areas with excessive access to (i.e., being swamped by) unhealthy food sources such as fast-food restaurants and corner stores [7,12,13]. Some studies have also considered the associations between different dietary environments and socioeconomic status to explore further whether there are inequalities in food access among sub-populations [14,15,16].

Previous research has proposed various recommendations on how to improve the food environment. The most common suggestion is that the government and society should provide support to improve the physical access in communities with poor grocery shopping or dietary environments. For example, to mitigate the food desert issue, it is often recommended that the government should encourage the opening of new businesses (such as supermarkets and grocery stores) through tax benefits and specific zoning policies [10,17,18]. On the other hand, in the case of food swamps, the government is urged to restrict relevant businesses such as fast-food restaurants [7,18,19].

So far, no research has specifically quantified people’s willingness to pay (WTP) for improving the dietary environment. Such work is essential and can help answer critical questions such as the following: Are residents living in food deserts willing to pay for better access to fresh food? If so, how much are they ready to pay? How much are people willing to spend to keep those fast-food restaurants away from their communities? These questions are crucial for estimating the economic benefits/costs of improving the food environment and guiding policy actions. To fill the research gap, this study aims to investigate people’s WTP for different types of food environments by utilizing the hedonic pricing model (HPM) to estimate the impact of diet environments on property values.

Given the unique distribution of food stores and increasing policy attention on food environments from the local government [11,13], this study takes the Canadian city of Edmonton as an illustration. Since a decade ago, the City of Edmonton has been making great efforts to build healthier and more secure food environments for Edmontonians, such as launching the city’s food and agriculture strategy, the *Fresh* [11,13]. This study focuses on examining people’s preferences for different food environments, assisting the local government in targeting the most vulnerable neighborhoods and developing tailored strategies to improve the diet environment.

The remainder of this article is structured as follows. Section 2 discusses empirical methods, including the way of determining the food environments, the introduction of the hedonic pricing models, the spatial regression model used to solve the spatial autocorrelation problem, and the calculation of the marginal effects of the spatial hedonic models. Section 3 describes the data and variable constructions. Section 4 presents the results and discusses the main findings. Section 5 concludes the article, provides the policy implications, and discusses directions for future research.

## 2. Methods

### 2.1. Food Environments

**Figure 1:**
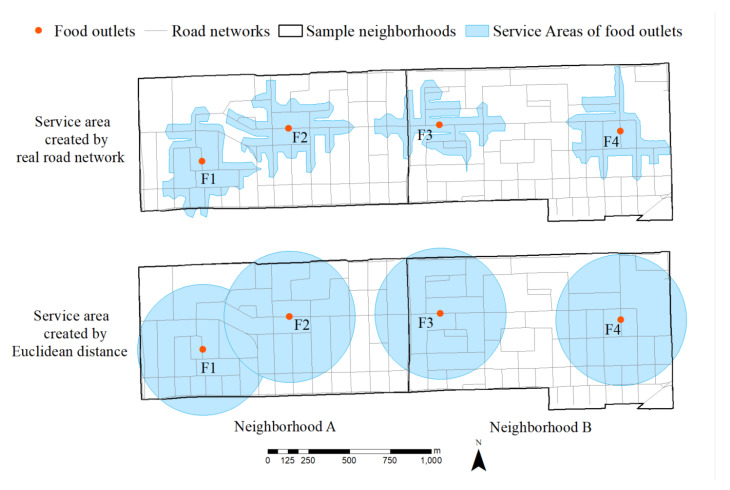
An illustration of service areas using road network and radius.

Following previous studies on identifying different types of food environments (e.g., [12,13]), this study uses the service area method to measure geographic access to healthy and unhealthy food outlets. A service area is a geographic area that includes all households that a specific food store can serve. In other words, all houses located in a service area can easily access the associated store for grocery shopping or dining. A simple illustration of the service area is presented in Figure 1. This study randomly selected two adjacent neighborhoods A and B from the study area. Both Neighborhoods A and B have two food stores, F1 and F2, and F3 and F4. In the upper panel, the blue zones around each store represent the service area defined using a road network distance of 400-m. It is clear from Figure 1 that although Neighborhood A has only two stores within the border, its residents along the edge (with Neighborhood B) can also shop from Food Store 3 in Neighborhood B. If this study only counts the number of stores, this edge effect will be ignored, thus underestimating the food environment in Neighborhood A. In the empirical analysis, this study uses road network distances instead of straight-line (Euclidean) distances, as many existing works do. To show the difference between the road network and the straight-line distances, a straight-line case is presented in the lower panel of Figure 1. The straight-line approach overestimates the serviceable areas and distorts the actual traveling distance.

### 2.2. The Spatial Hedonic Pricing Model

A general form of HPM can be expressed as 

$P_{i}=f\left(X_{i}\right)=f\left(F_{i},S_{i},L_{i},N_{i}\right)$, where $P_{i}$ represents the price of house *i* and $F_{i},S_{i},L_{i},\;N_{i}$ represent house *i*’s food environments, structural characteristics, locational characteristics, and neighborhood socioeconomic characteristics, respectively. Although some very few studies have employed HPM to assess the effect of proximity to supermarkets, grocery stores, and farmers’ markets on house prices (e.g., [20,21,22]), distance to a store and the overall community food environment are two very different concepts. The former cannot represent the latter. Researchers have used various functional forms to estimate HPM, including linear, log-log, and semi-log [23,24]. This study estimates the above functional forms and selects the one with the lowest Akaike information criterion (AIC) value.

**Figure 2:**
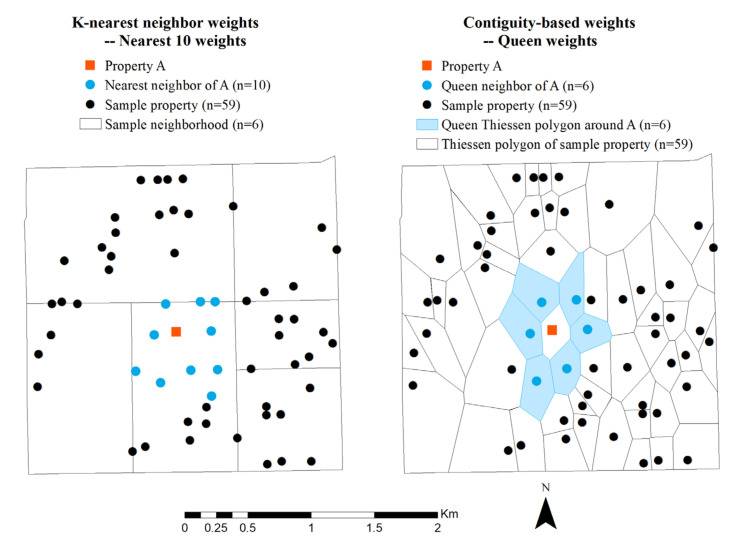
An illustration of the k-nearest and the queen neighbor definitions.

This study employs a spatial lag regression (SAR) model, which allows for spatial interactions in model specification to deal with the potential bias estimation problem. A SAR model can be expressed in a general form as:

(1) $$P=\alpha \iota_{n}+\rho WP+X\beta +\varepsilon ,\;\varepsilon \sim \left(0,\sigma^{2}I_{n}\right)$$

where *P* is an n×1 vector of the housing prices, $\iota_{n}$ and α represent the constant term and the associated coefficient, ρ is the spatial autoregressive coefficient, *W* is an n×n spatial weights matrix. This study considers both the *k*-nearest criterion (*k* = 5, 10) and the contiguity-based queen criterion to determine neighbor relationships. Figure 2 illustrates the neighbor definitions based on the two weights matrices. The term *WP* represents the weighted average of the housing prices from neighboring locations. The $X=\left(F,S,L,N\right)$ is an n×k matrix of observations on explanatory variables as defined in the previous subsection, β is a k×1 vector that represents parameters of explanatory variables, and ε is an n×1 vector of independent and identically distributed error terms.

### 2.3. The Marginal Effects and WTP in the SAR Model

Since presenting marginal effects in a matrix format is troublesome, this study follows LeSage and Pace (2009) [25] and reports the scalar summaries of average marginal effects. For each explanatory variable, this study reports the average direct effect (ADE), the average indirect effect (AIE), and the average total effect (ATE), which is the sum of ADE and AIE.

Knowing the average value of marginal effects, this study can further calculate the average WTP for living in different types of food environments. In terms of the log-log form hedonic model (the one with the lowest AIC in this case), the total, direct, and indirect WTP for dummy variables (e.g., whether living in a food swamp neighborhood) can be expressed as follows:

(2) $$Total\;WTP_{dummy}=\left[\exp(ATE)-1\right]\bar{P}\;Direct\;WTP_{dummy}=\left[\exp(ADE)-1\right]\bar{P}\;Indirect\;WTP_{dummy}=\left[\exp(AIE)-1\right]\bar{P}$$

where $\bar{P}$ represents the average value of properties. The calculation of WTP for continuous variables such as distance to the *River* and can be expressed as:

(3) $$Total\;WTP_{continuous}=ATE\frac{\bar{P}}{{\bar{x}}_{r}}\;Direct\;WTP_{continuous}=ADE\frac{\bar{P}}{{\bar{x}}_{r}}\;Indirect\;WTP_{continuous}=AIE\frac{\bar{P}}{{\bar{x}}_{r}}$$

where ${\bar{x}}_{r}$ represents the mean value of the explanatory variable *r*.

## 3. Data and Variable Construction

This study is conducted in Edmonton, Canada. Data for the empirical analysis are obtained from multiple sources. Table 1 presents the definitions and summary statistics for the variables. Housing transaction data for single-family residential properties are obtained from the RPS Real Property Solutions, covering the 2016–2017 period. This study obtains the location information of food outlets from the City of Edmonton business licenses database (2018) [26]. This database provided a list of establishments with a valid business license to operate in 2018. This study extracts location and other relevant information for supermarkets, local grocery stores, fast-food restaurants, and convenience stores to further analyze food environments. The business database contains the latitude and longitude of each business location, allowing us to geocode each food store. To check the accuracy of the food store data, every store information is compared with its official website, google map, and food review website. After the cross-comparison, the final food outlets dataset includes 91 supermarkets, 87 local grocery stores, 822 fast-food restaurants, and 232 convenience stores.

Following the practice of previous studies [7,12,15], this study defines supermarkets and local grocery stores as healthy food retailers and fast-food restaurants and convenience stores as unhealthy food outlets. Supermarkets and local grocery stores are categorized as healthy food outlets since they offer a wide variety of healthy foods such as fresh vegetables, fruits, dairy products, and meat [12,15]. On the other hand, fast-food restaurants and convenience stores are classified as unhealthy food outlets since they offer limited menu items and mainly provide processed or semi-processed foods with high energy density [7,12,15].

To identify the food environments in Edmonton, this study first follows previous studies (e.g., [13,17]) and adopts 1000-m (around 10–15 min walk) as the threshold to create service areas around each food outlet. Then, this study counts the food service areas for each neighborhood and defines three types of food environments based on the corresponding service-area counts. In particular, this study identifies type 1 food environment as neighborhoods with less than two service areas of supermarkets and grocery stores. This type of food environment represents neighborhoods in the bottom quantile of access to supermarkets and grocery stores and is similar to the concept of *food desert*. Similarly, this study identifies type 2 food environment as neighborhoods with more than four (i.e., the top quantile of) service areas of supermarkets and grocery stores. The type 2 environment is relevant to the concept of *food oasis* in the literature, indicating that communities have superior access to healthy food stores. Finally, this study labels type 3 food environment as neighborhoods with more than 23 (i.e., the top quantile of) service areas of fast-food restaurants and convenience stores. The type 3 food environment is relevant to the definition of *food swamp* in the literature, which means that these communities have too much exposure (i.e., been swamped) to unhealthy food retailers. Note that this study defines the food environments solely based on the access to different types of food stores. The literature, the definitions of food deserts, food swamps, and food oases usually also involve imposing relevant socioeconomic criteria, such as low income and high population density. This study takes such socioeconomic characteristics as control variables in the modeling procedure.

It is common to include houses’ locational attributes in the hedonic analysis since access to public services and amenities can significantly impact property values [27,28,29]. In the analysis, this study measures each house’s access to the North Saskatchewan River, Downtown Edmonton, the University of Alberta, and the nearest hospitals by calculating the distances between them using real road network data. The road network data of Edmonton and the locations of hospitals are retrieved from DMTI Spatial (2013) [30]. The shapefile of the North Saskatchewan River is obtained from the Government of Canada (2017) [31]. The size-based measurement is adopted to investigate the impacts of parks on house prices. Specifically, this study creates a 200 m buffer around each property and then computes the total square meters of parks within each buffer. The locations of parks (including neighborhood parks, community parks, city parks, and natural reserves), Downtown, and the University are obtained from the City of Edmonton Open Data Catalogue (2021a, 2021b) [32,33]. Socioeconomic variables are constructed based on the census data obtained from the City of Edmonton Open Data Catalogue (2018) [34]. A dummy variable to control the seasonal effects in the Edmonton housing market is also included.

## 4. Results and Discussion

### 4.1. Identification of Different Types of Food Environments

**Figure 3:**
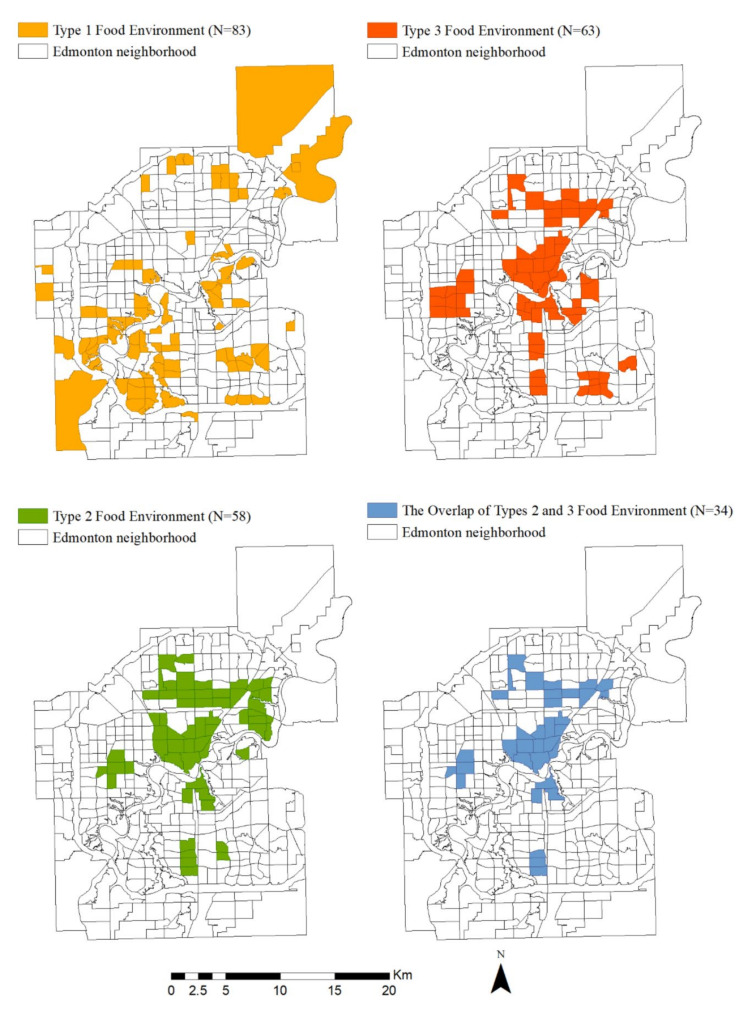
Identification of neighborhoods with different types of food environments.

This study identifies 83, 58, and 63 neighborhoods as type 1, type 2, and type 3, respectively. Furthermore, 34 neighborhoods are identified as overlaps of types 2 and 3, and no overlaps between types 1 and 2 or types 1 and 3. Figure 3 shows the distributions of the three types of neighborhoods in Edmonton. The type 1 neighborhoods (i.e., neighborhoods lack access to healthy food retailers) are clustered in the southwest of the city and scattered across the city fringe. There is a clear cluster in the north of the city for the type 2 neighborhoods (i.e., neighborhoods with superior access to healthy food retailers). The type 3 neighborhoods (i.e., neighborhoods with excessive access to unhealthy food outlets) are clustered in several parts of the city, including the city core, the university area, the northern area, the western area, and the southeast area. The overlaps of types 2 and 3 seem to be located mainly in the city core.

### 4.2. Estimation Results of Spatial Regressions

Before estimating the spatial regression models, the Moran’s I and the Lagrange Multiplier tests using the three different weights matrices (nearest 5 weights, nearest 10 weights, queen weights) are conducted. The results are presented in Table 2. The results confirm spatial autocorrelation and provide evidence for adopting the SAR model.

Table 3 presents the regression estimation results from the OLS model and the three SAR models with different weights matrices. The estimated spatial coefficients ρ in SAR models are all significant and positive. Due to the existence of spatial autocorrelation, the OLS results are biased. Therefore, this study focuses on the SAR results in the following discussion. Overall, the estimated signs of coefficients remain stable across the three SAR models. Among them, the SAR with the nearest-10 weights has the lowest AIC. The marginal effects and willingness to pay are thus derived based on this model.

This study reports the ADE, AIE, and ATE based on the nearest-10 weights in Table 4. The results indicate that house values increase by 0.83% if they locate in type 1 neighborhoods. The significant positive indirect effect (0.37%) suggests the type 1 neighborhood also exhibits positive externalities on nearby property values. The results further show that if a house is located in type 3 neighborhood, its value will increase by 2.27%, and the nearby house values will increase by 1.01%. Whereas, if a house is located in an overlap neighborhood, its price and the prices of nearby houses will decrease by 2.10% and 0.94%, respectively.

In terms of locational variables, the results show that when the distance between a house and the *River* decreases by 1%, its price increases by 2.11%, and all of the nearby house values increase by 0.94%. Besides, house price will increase by 16.09%, and all of the nearby house prices will increase by 7.18% if the distance between the house and the *University* decrease by 1%. The results also show that if a property has one square meter more of park area within its 200-m buffer, its price and the prices of nearby houses will increase significantly by 0.29% and 0.13%.

The corresponding WTP is presented in Table 5. The results indicate that for a house located in a type 1 neighborhood, the household is willing to pay C$5560.89 to reside in, and the nearby families are willing to pay a total of C$2471.24 to reside nearby. In a type 3 neighborhood, a household is willing to pay C$15,349.38 to live in this neighborhood. The neighboring households are willing to pay C$6781.37 to reside nearby. In addition, a household is willing to pay C$13,175.73 to live outside an overlap (of types 2 and 3) neighborhood, and the nearby residents are willing to pay a total of C$6070.56 to reside away from the overlap.

Concerning locational variables, the findings show that a household is willing to pay C$327.77 and C$942.10 for every 100-m decrease in distance to the *River* and the *University*, and the nearby residents are willing to pay C$146.15 and C$420.08 for every 100-m closer to the *River* and the *University*. Besides, a household is willing to pay C$44.52 for every 100 square meters of park area increase within the house’s 200-m buffer, and the nearby households are willing to pay C$19.85 to live nearby.

### 4.3. Discussion of the Estimation Results

The results show that people are willing to pay premiums to live in places with less healthy dietary environments. On the one hand, households are willing to pay extra to live in a place with limited access to supermarkets and grocery stores. On the other hand, people are willing to spend additional costs to live in an area with excess access to fast-food restaurants and convenience stores. From a healthy diet perspective, neither food deserts nor food swamps are ideal living environments. If he/she cares about health, a rational person will make the opposite choice. The media, diet experts, and scholars from various fields continually urge the government to improve residents’ grocery-shopping environment. The government should encourage the opening of new stores in desert neighborhoods and limit the fast-food restaurants in swamp communities to provide people with a better shopping environment and assist them in making better food choices. However, if people do not value a healthier eating environment, does the goodwill of the media and the advice of experts still apply?

Why would a rational person prefer to live in an unpleasant environment? As discussed in the introduction, the main reason is the multiple benefits and costs associated with different food environments. Supermarkets and fast-food restaurants are not just representatives of the eating environment. These stores also reflect other aspects related to people’s needs and preferences. For example, type 1 neighborhoods mean no supermarkets or grocery stores in the immediate living environment. The choice of residing in such an environment may reflect people’s dislike of a mixed (residential and commercial uses) land-use practice [17]. Since large supermarkets and grocery stores often bring negative impacts such as traffic jams, noise, lots of public visitors, and potential safety concerns [35,36]. Although people may also like the convenience of grocery shopping on foot, compared with the negative impacts these stores may bring, people would instead choose to live far away from these stores. This situation is particularly true in cities and towns in developed countries in North America. After all, individuals here only do grocery shopping approximately once a week on average [37,38] and most families have private cars, which makes it easy to drive to do shopping [39]. This reasonably explains why, on average, people have a positive WTP for the type 1 environment.

Similarly, people’s preference for type 3 environment does not necessarily come from people’s ignorance of health. Positive WTP may reflect people’s willingness to pay for convenience and time-saving. With raised income in modern society, the opportunity cost of cooking and housework is also increasing. Many families, especially dual workers, may not be willing or have time to cook after a day of work [40,41]. Fast food has become a convenient and affordable option. People can use the time saved to work, relax, socialize, play with children, etc. These can be counted as associated benefits/values brought by fast-food consumption. Therefore, people are not irrational about the positive WTP of living in the food-swamp type environment; it primarily reflects the preference of modern society for convenience and time-saving.

Another possible reason for preferring to live in type 3 environment is the low income. There will inevitably be low-income people and families who struggle with their daily expenses in any society. Around the world, healthy foods such as fresh vegetables and fruits are generally more expensive than energy-dense foods such as added-sugar and added-fats [42,43,44]. Not counting the extra time for shopping, cooking, and packing, only considering the price aspect, daily consumption of fresh fruits and vegetables exceeds the budget of low-income families. For example, in 2017–2018, one in eight households (approximately 4.4 million people) in Canada experienced food insecurity. That means these households lacked sufficient financial resources to provide enough food for all family members at some point of the year [45]. For such families, fast food seems to be a wise choice. Therefore, many low-income families may be willing to pay extra costs in exchange for living in areas with a convenient fast-food environment.

## 5. Conclusions

This study estimates people’s WTP for different types of food environments using spatial hedonic pricing models. The empirical results show that people are willing to pay a premium to live in type 1 (food-desert type) and type 3 (food-swamp type) environments. Why do rational people prefer to live in disadvantaged food environments? Several potential reasons are discussed to explain such seemingly irrational behaviors. The premium paid to live in type 1 environment may reflect people’s dislike of noise, traffic jams, and potential safety issues brought by supermarkets and grocery stores. The WTP for living in type 3 environment may reflect people’s preference for convenience and time-saving brought by fast-food consumption in modern urban society. Additionally, the inability of low-income families to afford healthy food may be a deeper reason why they choose to live in neighborhoods with excess access to fast food.

Based on the result that people are willing to pay a premium to live in type 1 environment, the government’s strategy of encouraging new stores in the community to improve the food environment may not be the most effective method. Doing so will reduce many residents’ utility (i.e., the overall happiness or satisfaction) of many residents. Obviously not everyone wants to have a supermarket or grocery store in his community. Such new businesses may also affect local government revenue since the negative WTP value will be reflected in housing prices. Local taxes based on housing prices are a significant part of municipal revenue. Of course, not all families do not welcome supermarkets in their community. Some families may not own a vehicle, do not have easy access to public transportation, or cannot afford to go to a distant supermarket for shopping. This group of residents will need help from the government and society.

In response to the situation that people prefer a type 3 environment due to the benefits associated with fast-food consumption, policies can encourage healthier fast-food restaurants, such as salad bars and green smoothie shops. The key is to promote restaurants that combine healthy ingredients and quick servicing time. Transform the traditional fast-food industry of high calorie, high sugar, and high carbohydrate into a healthy and fast new fast-food model.

To solve the situation that people are willing to live in type 3 environment due to low income, the government can provide free grocery shopping vouchers and subsidize public transportation tickets for grocery shopping. Besides, many cities in Canada, including Edmonton, have community garden projects. Harvested vegetables and fruits in community gardens can be shared among members or families in the community and can be sold at local farmers’ markets to earn additional income [46]. Such projects shall continue to be promoted, and the government and society can further help the communities, especially the low-income group, by subsidizing production inputs and contributing voluntary labor.

In addition to demand/consumer considerations, a specific food environment is also highly related to the supply side. After all, food availability is a result of business location decisions. Studies have shown that in recent decades, the suburbanization of North American food retailers has contributed to the emergence of urban food deserts/swamps or disadvantaged urban areas where it is relatively difficult to obtain healthy and affordable food (e.g., [17,47]). Policies to improve public health and the built environment must also recognize the potential impact of suburbanization on access to healthy and affordable food. To better understand how food availability affects consumer behavior and related health outcomes, it is necessary to conduct other research from the food supply side (for example, business location determination) in the context of urban sprawl and increasing developmental pressures.

This study has some limitations, and these gaps would benefit from further research. First, this study did not consider the sorting issue [48,49], which might lead to estimation bias. Households may self-select into specific food environments (e.g., low-income households may choose to live in type 3 neighborhoods), and the unobserved error term in the location decision may be associated with one or more missing variables in the hedonic price equation. To deal with the selection issue, future research may consider employing a spatial sorting model that allows unobserved error terms in neighborhood selection to be correlated with unobserved errors in the hedonic equation. Second, when investigating health food stores, this study focused on supermarkets and large grocery stores that offer a variety of healthy food items. On the one hand, local food systems (e.g., farmers’ markets, community gardens, and community-supported agriculture) have played an important role in increasing the availability of healthy food options [11,21]. On the other hand, with an increasing demand for fresh and nutritious food, local food systems also bring environmental and social benefits that significantly impact residents’ WTP [11,21]. Future research should find it helpful to include such local food establishments in food environment assessments and WTP estimations.

## Figures and Tables

**Table 1:** Variable Definitions and Summary Statistics (n = 4398).

Variables	Definition	Mean	Std. Dev.
Dependent Variable			
*Price ^a^*	Sale price of the property (2016$)	454,882.50	201,652.30
Food Environment Types			
*Type 1*	1 if house is located in a type 1 neighborhood, 0 otherwise	0.33	0.47
*Type 2*	1 if house is located in a type 2 neighborhood, 0 otherwise	0.21	0.41
*Type 3*	1 if house is located in a Type 3 neighborhood, 0 otherwise	0.21	0.41
*The overlap of Types 2 and 3*	1 if house is located in the overlap of a types 2 and 3, 0 otherwise	0.12	0.32
Structural Variables			
*Living area ^a^*	Square feet of living spaces	1553.53	607.44
*Lot size ^a^*	Square feet of lands owned by a household	5939.27	5351.73
*Bedroom*	Number of bedrooms	2.91	0.65
*Bathroom*	Number of bathrooms	1.62	0.66
*Basement condition*	3 if the basement is finished, 2 if the basement is partial finished, and 1 if the basement is unfinished	2.47	0.81
*House condition*	4 if the house condition is excellent, 3 if the house condition is good, 2 if the house condition is average, and 1 if the house condition is poor	3.02	0.85
*Garage*	Capacity of garages (double or single)	1.84	0.47
*House age*	Age of the house	27.93	22.59
Locational Variables			
*River ^a^*	Distance to North Saskatchewan River	4281.59	3248.10
*Downtown ^a^*	Distance to Downtown	10,503.78	4311.75
*University ^a^*	Distance to University of Alberta	11,351.15	3957.81
*Hospital ^a^*	Distance to the nearest hospital	5049.93	2369.12
*Park ^a^*	m^2^ of park within a 200-m buffer	4274.69	9590.85
Neighborhood Socio-economic Status			
*Population density ^a^*	Neighborhood level population density (Per capita/Km^2^)	3063.59	1054.32
*Children*	The ratio of the children aged under 14	0.18	0.05
*Senior*	The ratio of the senior population aged over 65	0.14	0.08
*High education*	The ratio of residents who have a postsecondary certificate, diploma, or degree	0.63	0.12
*Unemployment*	The ratio of residents who are unemployed	0.09	0.04
*Low income*	The ratio of residents who have a relative low income (annual income less than C$30,000)	0.13	0.10
*High income*	The ratio of residents who have a relative high income (annual income more than C$150,000)	0.17	0.12
*Season*	1 if house is sold between April and September, 0 otherwise	0.55	0.50

*^a^* In the method and empirical section, these variables are transformed to log forms.

**Table 2:** Tests for Spatial Dependence.

		K-Nearest Neighbor Weights (Nearest 5)	K-Nearest Neighbor Weights (Nearest 10)	Contiguity-Based Weights (First Order Queen)
Moran’s I	Statistic	0.244	0.221	0.242
	*p*-value	2.20 × 10^−16^	2.20 × 10^−16^	2.20 × 10^−16^
LM spatial lag	Statistic	495.170	606.510	537.700
	*p*-value	2.20 × 10^−16^	2.20 × 10^−16^	2.20 × 10^−16^
Robust LM spatial lag	Statistic	71.774	84.638	83.965
	*p*-value	2.20 × 10^−16^	2.20 × 10^−16^	2.20 × 10^−16^

**Table 3:** Estimation Results of Different Hedonic Models.

Variables	OLS Model	SAR		
		Nearest 5 Weights	Nearest 10 Weights	Queen Weights
Food Environment Types				
*Type 1*	0.014 ***	0.008	0.008 *	0.008 *
	(0.005)	(0.005)	(0.005)	(0.005)
*Type 2*	−0.014	−0.012	−0.012	−0.009
	(0.009)	(0.008)	(0.008)	(0.008)
*Type 3*	0.037 ***	0.025 ***	0.022 ***	0.026 ***
	(0.008)	(0.008)	(0.008)	(0.008)
*The overlap of Types 2 and 3*	−0.047 ***	−0.037 ***	−0.031 ***	−0.038 ***
	(0.012)	(0.012)	(0.012)	(0.012)
Structural Variables				
*Log (Living area)*	0.591 ***	0.532 ***	0.534 ***	0.532 ***
	(0.011)	(0.011)	(0.011)	(0.011)
*Log (Lot size)*	0.091 ***	0.080 ***	0.081 ***	0.081 ***
	(0.006)	(0.005)	(0.005)	(0.005)
*Bedroom*	−0.051 ***	−0.043 ***	−0.043 ***	−0.042 ***
	(0.004)	(0.004)	(0.004)	(0.004)
*Bathroom*	0.023 ***	0.020 ***	0.020 ***	0.021 ***
	(0.005)	(0.004)	(0.004)	(0.004)
*House condition*	0.013 ***	0.012 ***	0.012 ***	0.013 ***
	(0.003)	(0.002)	(0.002)	(0.002)
*Basement condition*	0.048 ***	0.046 ***	0.047 ***	0.046 ***
	(0.003)	(0.003)	(0.003)	(0.003)
*Garage*	0.102 ***	0.092 ***	0.093 ***	0.094 ***
	(0.005)	(0.005)	(0.005)	(0.005)
*House age*	−0.003 ***	−0.003 ***	−0.003 ***	−0.003 ***
	(0.000)	(0.000)	(0.000)	(0.000)
*House age^2^*	0.000 ***	0.000 ***	0.000 ***	0.000 ***
	(0.000)	(0.000)	(0.000)	(0.000)
Locational Variables				
*Log (River)*	−0.030 ***	−0.024 ***	−0.021 ***	−0.021 ***
	(0.003)	(0.003)	(0.003)	(0.003)
*Log (Downtown)*	0.014	−0.004	−0.015	−0.013
	(0.012)	(0.011)	(0.011)	(0.011)
*Log (University)*	−0.224 ***	−0.171 ***	−0.159 ***	−0.168 ***
	(0.013)	(0.012)	(0.012)	(0.012)
*Log (Hospital)*	0.007	0.001	−0.003	−0.001
	(0.005)	(0.004)	(0.004)	(0.004)
*Log (Park)*	0.003 ***	0.003 ***	0.003 ***	0.002 ***
	(0.001)	(0.000)	(0.000)	(0.000)
Neighborhood Socio-economic Status				
*Log (Population density)*	−0.026 ***	−0.021 ***	−0.028 ***	−0.025 ***
	(0.007)	(0.007)	(0.006)	(0.006)
*Children*	0.282 ***	0.174 **	0.145 **	0.188 ***
	(0.077)	(0.072)	(0.072)	(0.072)
*Senior*	0.203 ***	0.171 ***	0.155 ***	0.168 ***
	(0.044)	(0.042)	(0.042)	(0.042)
*High education*	0.365 ***	0.209 ***	0.169 ***	0.203 ***
	(0.035)	(0.034)	(0.034)	(0.034)
*Unemployment*	−0.053	−0.082	−0.058	−0.070
	(0.106)	(0.100)	(0.100)	(0.100)
*Low income*	−0.212 ***	−0.173 ***	−0.189 ***	−0.197 ***
	(0.045)	(0.042)	(0.042)	(0.042)
*High income*	0.129 ***	−0.047	−0.084 **	−0.056
	(0.036)	(0.035)	(0.035)	(0.035)
*Season*	0.010 **	0.010 **	0.011 ***	0.011 ***
	(0.004)	(0.004)	(0.004)	(0.004)
Constant	9.705 ***	6.569 ***	6.007 ***	6.507 ***
	(0.132)	(0.186)	(0.198)	(0.191)
Adjusted R^2^	0.8437			
Rho		0.266 ***	0.3155 ***	0.2770 ***
Log Likelihood		2773.06	2794.62	2784.00
AIC		−5488.10	−5531.20	−5510.00

Note: Significance denoted by *** *p* < 0.01, ** *p* < 0.05, and * *p* < 0.1.

**Table 4:** Marginal Effects for Spatial Lag Model Based on Nearest 10 Weights.

Variables	ADE	AIE	ATE
Food Environment Types			
*Type 1*	0.0083 *	0.0037 *	0.0120 *
	(0.0050)	(0.0022)	(0.0072)
*Type 2*	−0.0126	−0.0056	−0.0182
	(0.0083)	(0.0037)	(0.0120)
*Type 3*	0.0227 ***	0.0101 ***	0.0328 ***
	(0.0078)	(0.0035)	(0.0112)
*The overlap of Types 2 and 3* ^a^	−0.0311 ***	−0.0139 ***	−0.0450 ***
	(0.0118)	(0.0053)	(0.0171)
Locational Variables			
*Log (River)*	−0.0211 ***	−0.0094 ***	−0.0305 ***
	(0.0029)	(0.0013)	(0.0042)
*Log (Downtown)*	−0.0153	−0.0068	−0.0222
	(0.0113)	(0.0051)	(0.0164)
*Log (University)*	−0.1609 ***	−0.0718 ***	−0.2327 ***
	(0.0124)	(0.0063)	(0.0176)
*Log (Hospital)*	−0.0031	−0.0014	−0.0045
	(0.0046)	(0.0021)	(0.0067)
*Log (Park)*	0.0029 ***	0.0013 ***	0.0041 ***
	(0.0005)	(0.0002)	(0.0007)

Note: ^a^ If a house is located in an overlap of types 2 and 3 neighborhood, its price and the prices of nearby houses will decrease by 2.10% (−1.26% + 2.27% − 3.11% = −2.10%) and 0.94% (−0.56% + 1.01% − 1.39% = −0.94%) when the dummy variables of *Type 2*, *Type 3*, and *The overlap of Types 2 and 3* all equal to 1. Significance denoted by *** *p* < 0.01, ** *p* < 0.05, and * *p* < 0.1.

**Table 5:** The WTP for Spatial Lag Model Based on Nearest 10 Weights.

Variables	WTP for OLS Model	WTP for SAR		
		Direct	Indirect	Total
Food Environment Types				
*Type 1*	6620.77 ***	5560.89 *	2471.24 *	8062.34 *
*Type 2*	−6193.88	−8296.47	−3718.25	−11,946.91
*Type 3*	17,325.30 ***	15,349.38 ***	6781.37 ***	22,359.57 ***
*The overlap of Types 2 and 3* ^a^	−20,884.84 ***	−20,228.64 ***	−9133.68 ***	−28,956.15 ***
Locational Variables				
*River* ^b^	315.43 ***	327.77 ***	146.15 ***	473.93 ***
*Downtown* ^b^	−58.94	96.96	43.23	140.19
*University* ^b^	897.25 ***	942.10 ***	420.08 ***	1362.18 ***
*Hospital* ^b^	−64.44	41.35	18.44	59.79
*Park* ^c^	33.13 ***	44.52 ***	19.85 ***	64.38 ***

Note: ^a^ If a house is located in an overlap of types 2 and 3 neighborhood, the household is willing to pay C$13,175.73 (−8296.47+15,349.38−20,228.64=−13,175.73) to keep away from the house when the dummy variables of *Type 2*, *Type 3*, and *The overlap of Types 2 and 3* all equal to 1. The nearby residents are willing to pay a total of C$6070.56 (−3718.25+6781.37−9133.68=−6070.56) to live away when the dummy variables of *Type 2, Type 3,* and *The overlap* all equal to 1. ^b^ The WTP estimation is based on people’s WTP for every 100-m decrease in distance to a certain amenity. ^c^ The WTP estimation is based on people’s WTP for every 100 square meters of park area increase within the house’s 200-m buffer. Significance denoted by *** *p* < 0.01, ** *p* < 0.05, and * *p* < 0.1.

## Data Availability

The data that support the findings of this study are available from RPS Real Property Solutions (RPS) but restrictions apply to the availability of these data, which were used under license for the current study, and so are not publicly available. Data are however available from the authors upon reasonable request and with permission of RPS.
